# Effects of an Afrocentric Sexual Health Education Curriculum for Medical, Nursing, and Midwifery Students in Tanzania: A Single-Blinded Randomized Controlled Phase 3 Trial

**DOI:** 10.1007/s10508-025-03207-1

**Published:** 2025-08-11

**Authors:** B. R. Simon Rosser, Dickson Ally Mkoka, Maria Trent, Nidhi Kohli, Lucy R. Mgopa, Corissa T. Rohloff, Ever Mkonyi, Michael W. Ross, Stella Emmanuel Mushy, Inari Mohammed, Agnes F. Massae, Ziwei Zhang, Dorkasi L. Mwakawanga, Gift Gadiel Lukumay

**Affiliations:** 1 Division of Epidemiology and Community Health, School of Public Health, University of Minnesota, 1300 S. 2nd St., #300, Minneapolis, MN 55454 USA; 2 Department of Community Health Nursing, School of Nursing, Muhimbili University of Health and Allied Sciences, Dar Es Salaam, Tanzania; 3 Division of Adolescent and Young Adult Medicine, Johns Hopkins University, Baltimore, MD USA; 4 Department of Educational Psychology, University of Minnesota, Minneapolis, MN USA; 5 Department of Psychiatry, School of Medicine, Muhimbili University of Health and Allied Sciences, Dar Es Salaam, Tanzania; 6 Eli Coleman Institute for Sexual and Gender Health, Department of Family Medicine at the University of Minnesota, Minneapolis, MN USA

**Keywords:** Sex education, East African People, Health care students

## Abstract

Sub-Saharan Africa has the world’s highest rates of sexual health challenges. Yet, sexual health curricula for health students are rare. To advance research on the effects of such a curricula, we conducted the first randomized controlled trial of a sexual health curriculum for health students. “Training for Health Professionals” was a randomized, controlled, single-blind, trial conducted in Tanzania. In 2021, 412 nursing, midwifery, and medical students were stratified by discipline, completed baseline assessments, then randomized to receive a four-day comprehensive curriculum (*n* = 206) or to a waitlist control (*n* = 206). The curriculum covered sexual health across the lifespan, male and female sexual dysfunctions, key populations (LGBT, sex workers), sexual violence, clinical skills building, ethics, policy writing, and cultural considerations. Primary outcomes were assessments of sexual health knowledge, attitudes, and clinical skills at baseline, post-intervention, and three-month follow-up. Clinical skills were evaluated through videotaped standardized patient interviews assessed by expert raters blind to arm of study or baseline/follow-up interview. Attrition was minimal (< 1%); final sample size was 408. Compared to control, intervention participants had statistically significant, moderate to large, increases in sexual health knowledge ($\beta =3.49, SE=0.24, p<0.001$), confidence in addressing patients’ concerns ($\beta =29.34, SE=3.26, p<0.001$), ability to discuss sexual health with patients ($\beta =22.00, SE=1.99, p<0.001$), and improved clinical skills ($\beta =8.04, SE=0.60,\,p<0.001$ for interpersonal communication; $\beta =2.50, SE=0.28, p<0.001$ for medical history taking). Most participants (76.6%) evaluated the curriculum as culturally appropriate for Africa. No adverse effects were observed. This study provides “gold standard” evidence that training in sexual health is culturally acceptable, needed, and effective for nursing, midwifery, and medical students. Such training may be particularly important in sub-Saharan Africa and low- and middle-income countries given substantial sexual health challenges.

## Introduction

Sexual health is “a state of physical, emotional, mental and social well-being in relation to sexuality; it is not merely the absence of disease, dysfunction or infirmity. Sexual health requires a positive and respectful approach to sexuality and sexual relationships, as well as the possibility of having pleasurable and safe sexual experiences, free of coercion, discrimination and violence. For sexual health to be attained and maintained, the sexual rights of all persons must be respected, protected and fulfilled” (World Health Organization, 2006). In high-income countries, such as the USA, sexual health is widely recognized as an “intrinsic element of human health,” (Douglas & Fenton, 2013; Office of the Surgeon General, 2001) and integrated into the nation’s health goals (National Academies of Sciences & Medicine, 2020). Despite this recognition, in the US, 50% of medical students state the training they received was unsatisfactory, including 34% who report there was no formal curriculum (Beebe et al., 2021; Kemble et al., 2023) while 27% of baccalaureate nursing programs report having no formal sexual health curriculum (Aaberg, 2016). While aspects of sexual health have been studied since the 1800s, the effects of sexual health training on clinicians have not been rigorously researched. To the best of our review, we could not find any prior randomized controlled studies of the effectiveness of sexual health curricula on nursing, midwifery or medical students. This has left educators to argue the pros and cons of such education without the benefit of rigorous studies detailing its effects on knowledge, attitudes and clinical skills.

Research on patients in the USA shows most prefer to receive sexual health information from their provider and for the provider to initiate this conversation (Wittenberg & Gerber, 2009). Most health students feel addressing and treating sexual health concerns will be an important part of their careers, yet only about a third feel adequately trained to do so (Wittenberg & Gerber, 2009). Medical students appear under-prepared to address patients’ sexual health concerns (Warner et al., 2018). In a national survey of sexual health knowledge of 1014 US medical students in the USA, most failed a passing rate in four of six knowledge categories (Warner et al., 2018). Similarly, surveys of nursing students in Turkey show that two-thirds of students do not feel comfortable discussing sexual issues with patients (Bal & Sahiner, 2015). In Taiwan, a study of 190 nursing students observed that improved sexual health knowledge is correlated with better attitudes toward sexual health care and confidence providing such care (Sung et al., 2015). Their key conclusion is that sexual health training of nurses needs to address both knowledge and trainee nurses’ attitudes toward sexual health.

Several evaluation studies of sexual education courses for medical students have been conducted mainly in the USA and Latin America, and almost all in the 1970s. While some found no effects but were not powered to show effects (Golden & Liston, 1972; Marcotte, 1973), most showed improvements in sexual knowledge and adoption of more tolerant attitudes toward sexual behaviors and/or patients presenting with sexual concerns (Alzate, 1982, 1990; Garrard et al., 1976; Marcotte & Kilpatrick, 1974; Marcotte & Logan, 1977; Rosenberg & Chilgren, 1973; Schnarch & Jones, 1981). The same effects have been reported for nursing students (Mims et al., 1976). However, the studies were all uncontrolled, response rates and attrition not reported, and most relied on pre-, immediate post-test quasi-experimental designs. In 2019, Ross et al. (2021) evaluated their sexual health course using data on 74 medical students in the USA. They found significant increases in knowledge and self-assessed communication skills with patients, but little change in attitudes about sexuality. Unfortunately, only 43% of students taking the course completed the evaluation making interpretation of findings difficult. Missing from the research is any rigorous, controlled, appropriate powered longitudinal studies using validated instruments including objective ratings of the effects of such education on clinical skills. This study is designed to address these gaps.

In low- and middle-income countries, where sexual and reproductive health challenges are often greatest, sexual health curricula to train medical, nursing and midwifery students are even more rare. No country in sub-Saharan Africa requires sexual health education for health students and we could identify only two health universities on the continent who currently teach a formal sexual health curriculum. The impact of this lack on patient care was highlighted in a recent study of patients with hypertension and diabetes in the North West province, South Africa (Pretorius, 2021). Sexual histories were taken in only 3% of cases, HIV risk was routinely not assessed, patients with sexual dysfunctions were missed, and patients presenting with sexual health concerns encountered provider paternalism and a lack of warmth and respect (Pretorius, 2021).

The United Republic of Tanzania has a population of 62 million, 63% of whom live in rural areas and 49% who live in poverty (< US$1.90 per day). With just 20,800 nurses, 2600 medical doctors and 739 specialists (Cowling, 2024), it has an acute shortage of healthcare workers. While WHO recommends a minimum of 22.8 clinicians and nurses per 10,000 populations, for Tanzania this ratio is just 8.42 per 10,000 population (World Bank, 2024). With the extremely low doctor-to-patient ratio, limited healthcare infrastructure and poverty being substantial structural barriers, most Tanzanians will not see a medical doctor in their lifetime (Peck, 2013). Tanzania is a socially conservative country (Stankov & Lee, 2016). For example, public displays of affection, including kissing, hugging or holding hands, are generally considered inappropriate (Grant, 2025), while the role of women is described as very conservative and patriarchal (Vyas & Jansen, 2018). Tanzania also has among the greatest sexual and reproductive health challenges of any country (Rosser et al., 2022). Rates of HIV (Tanzania Commission for AIDS, 2013), STIs (Aboud et al., 2023), unplanned teen pregnancy (Exavery et al., 2014) and sexual violence (García-Moreno et al., 2005; Mgopa 2017, 2021a, 2021b) are among the highest in sub-Saharan Africa and leading contributors to morbidity and mortality. Community myths about sexual healthcare and cultural taboos about discussing sex create further barriers to addressing patients’ concerns (Lukumay et al., 2023). Midwives, nurses and doctors provide the majority of health delivery in Tanzania. Without sexual health training, they report providing misinformation and improper care to patients (Mgopa et al., 2021a, 2021b; Mkonyi et al., 2021; Mushy et al., 2021; Mwakawanga et al., 2021).

The long-term objective of this study is to improve the sexual health of patients in Tanzania by training the health workforce in sexual health. The aim of this randomized controlled trial was to evaluate the effectiveness of an Afrocentric sexual health curriculum on medical, nursing and midwifery students’ knowledge, attitudes and clinical skills in providing sexual health care.

## Method

### Study Design

The Training for Health Professionals study was a stratified (by discipline), randomized controlled, single-blind parallel trial of a sexual health curriculum versus a waitlist control. To prevent discipline effects contaminating the study, randomization was stratified by discipline (nursing, midwifery and medical). The study was undertaken with students at the Muhimbili University of Health and Allied Sciences (MUHAS) in Dar Es Salaam, Tanzania.

### Participants

Eligibility criteria for this study were: (1) Current student at MUHAS in midwifery, nursing or medicine; (2) in their 3rd or 4th year (for medical students) or 2nd or 3rd year (nursing and midwifery students) so they would have sustained patient contact in the three months following the seminar and be on campus for the follow-up; (3) able to attend the full four-day training during the first week of student vacation; (4) fluent in English (the language of instruction at MUHAS) and Kiswahili (the *lingua franca* in Tanzania); and (5) willing to volunteer and complete all evaluation procedures. Students were recruited using flyers on campus noticeboards and from announcements in class. For COVID prevention, on each day of study procedures, all participants and staff had their temperature checked, masks were obligatory, and had assigned seats with spacing in the lecture theater. Anyone with a high temperature was excluded from participation. All participants provided signed informed consent prior to commencing baseline study activities.

**Fig. 1:**
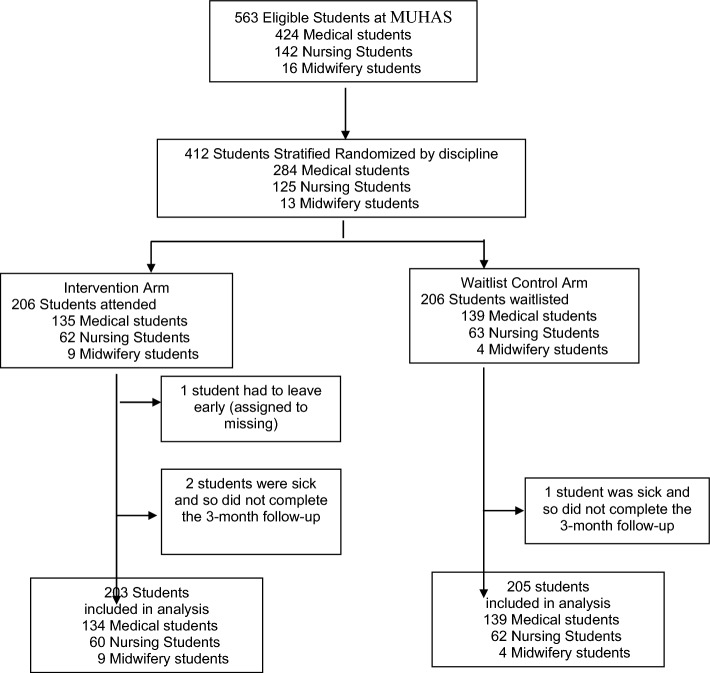
Trial profile

Between May 1, 2021 and July 31, 2021, participants were recruited for the current study until the target recruitment was reached (see Fig. 1 for trial profile). Of 563 students who were potentially eligible, the first 412 participants were recruited, underwent an informed consent process, completed baseline data collection and randomized into intervention (*n* = 206) and control (*n* = 206) groups. Table 1 presents the demographic information of the intervention and control group participants at baseline.

**Table 1:** Baseline demographic characteristics of the Intervention and Control Groups

	Control (*N* = 206)		Intervention (*N* = 206)	
	*n*	*%*	*n*	*%*
Discipline				
Medical	139	67.5	135	65.5
Nursing	63	30.6	62	30.1
Midwifery	4	1.9	9	4.4
Year of study				
Final	102	49.5	99	48.1
Penultimate	104	50.5	107	51.9
Gender				
Female	62	30.1	64	31.1
Male	139	67.5	137	66.5
Other/prefer not to answer	5	2.4	5	2.4
Age (M, SD)	24.0	2.5	23.9	2.3
Relationship status				
Single	188	91.3	194	94.2
Married (monogamously)	10		7	3.4
Cohabitating	5	2.4	3	1.5
Other/prefer not to answer	3	1.5	2	1.0
Religious affiliation				
Christian	174	84.5	175	85.0
Muslim	29	14.1	30	14.6
Other/Prefer not to answer	3	1.5	1	0.5
Religiosity				
Not at all religious	2	1.0	1	0.5
Slightly religious	6	2.9	11	5.3
Moderately religious	125	60.7	124	60.2
Very religious	51	24.8	58	
Extremely religious	10	4.9	11	5.3
Prefer not to answer	12	5.8	1	0.5
Did you receive sexual health education prior to attending MUHAS?				
Yes	121	58.7	118	57.3
No	64	31.1	68	33.0
Unsure/Don’t remember	21	10.2	20	9.7

Age (M, SD) = using mean and standard deviation to summarize age rather than using *n* and %

#### Randomization and Masking

Project staff kept three envelopes titled “midwifery,” “nursing” and “medical” students. Inside each envelope were 10 pieces of paper, five labeled “2021,” and five labeled “2022.” If a participant drew a piece of paper with “2021” on it, they were informed they would attend the training in 2021 (intervention group). Alternatively, those who drew a piece of paper with “2022” on it, were informed they would attend the training in 2022 (waitlist control group).

Once a piece of paper was drawn, it was not returned to the envelope but instead, put in the “used envelope” for reuse later. Thus, the first midwifery student drew from an envelope with ten pieces of paper in it, the second with nine and so on. Once the envelope was empty, it was refilled. Study staff recorded the randomization (to prevent participants from changing their assignment). To minimize expectation effects, staff were trained to only refer to the assignment by year and not to use the terms “intervention” or “control.”

The study coordinator gave the participants assigned to the 2021 training a one-page flyer providing the participant with all the information about the seminar including that the participant was expected to attend all days. Participants assigned to 2022 were given a different one-page flyer that thanked them for completing the baseline assessment survey and standardized patient interviews, informed them they would be asked to complete another assessment in three to four months’ time, and told they could attend the training in September 2022. Both flyers had a sentence noting that participation in the research study was voluntary and that the participant could withdraw from the study at any time.

To assess the clinical skills of students, this was a single-blind study of the videotaped standardized patient interviews. At analysis, all videos were assigned a code and randomized so that the evaluators would not know whether the interview was from the pre-test or follow-up or whether the student was in the intervention or control group.

### Procedure

All participants were required to complete the online Qualtrics baseline survey on tablets at the study office. The survey took about 60 min to complete and covered demographic information, educational background, sexual health knowledge and attitudes toward sexual health topics. After completing the survey, participants completed two standardized patient interviews. The interviews involved actors role-playing one of four sexual health-related patient scenarios developed by the team. Participants had 10 min per scenario to interview the patient and discuss a treatment plan. The four standardized patient cases were: (1) a woman who had been physically and sexually assaulted, (2) a heterosexually married man with situational erectile dysfunction and past homosexual experience, (3) a 16-year-old girl who is worried she is pregnant from her older “sugar daddy” boyfriend, and (4) a young heterosexual man with penile discharge, groin pain and a history of paying for sex. The participants were randomly assigned two of the four scenarios which they completed at baseline. Then, they completed the remaining two scenarios at follow-up. Participants who completed all baseline procedures were compensated TZS110,000 ($50).

#### Intervention

The intervention was a four-day, Afrocentric, comprehensive sexual health curriculum, and described in detail elsewhere (Mkoka et al., 2025). To summarize, the pilot curriculum was based on one developed by the senior author for the Pan American Health Organization/World Health Organization. To deeply embed it in the African context, we reviewed the sexual health literature and epidemiology from Africa and used local statistics (e.g., HIV and STIs in Tanzania) wherever available. In addition, we conducted 18 focus groups with clinicians and students in Tanzania to identify local sexual health concerns (Rosser et al., 2022), clinical practices (Mgopa et al., 2021a, 2021b, 2022; Mkonyi et al., 2021; Mushy et al., 2021; Mwakawanga et al., 2021), and we interviewed 11 community leaders to identify barriers, common myths and misconceptions (Lukumay et al., 2023). Then, we rewrote the curriculum to focus on the most common sexual health challenges clinicians experience in Tanzania. To further tailor it, all materials and exercises were translated to make the curriculum fully bilingual (in English and Kiswahili) and all modules were written and delivered by Tanzanian faculty. We knew from our formative research that 81% of midwifery, 89% of nursing and 73% of medical students stated they would prefer to learn together so we developed one curriculum deliverable across the three disciplines rather than separate curricula tailored to each discipline (Rosser et al., 2022). The curriculum covered sexual health across the lifespan and male and female sexual dysfunctions (Day 1); key populations (LGBT, sex workers) and sexual violence (Day 2); clinical skills training, ethics and policy writing (Day 3); and community resources and cultural considerations (Day 4). To maximize relevance for health students training to be clinicians, Day 3 was framed as a clinical skills “boot camp” for them to practice sexual health history taking, counseling, ethical decision-making and policy writing. Immediately after the training, intervention arm participants completed an online post-test survey programmed in Qualtrics that measured changes in sexual health knowledge and attitudes toward sexual health topics and asked participants to evaluate the sexual health training (90 min).

Three to four months later, both intervention and control group participants completed an online follow-up survey that assessed their sexual health knowledge and attitudes toward sexual health topics (90 min) and the remaining two videotaped standardized patient scenarios (10 min each). Participants then completed a short evaluation of the standardized patient activities, and intervention group participants a short survey to assess their evaluation of the sexual health curriculum three to four months after completing the training. All online survey responses were saved automatically in the Qualtrics database upon participants’ completion. Two research staff monitored the database and ensured that all survey data had been recorded and completed.

### Measures

#### Demographic Variables

Age was asked in years, gender as male, female or “other, specify” and region of Tanzania where they were born and raised as two questions with write-in responses. If people were born or raised outside of Tanzania, they were asked to put in the country of the birth or where they were raised. Relations status was asked as “What is your relationship status? With responses being single, cohabitating, married to one partner (monogamy), married to more than one partner (polygamy) divorces separated and widowed.” Religion was asked as “what is your religion” with seven response options reflecting the six most common affiliations (i.e., Buddhist, Christian, Muslim, Hindu Paganist and no religion) plus a write-in option of another religion. The next question asked, “How religious are you” on a 5 point scale from “1 = not at all religious” to “5 = extremely religiously.” In addition, all items had a “prefer not to answer” option to remind participants that responses were voluntary.

#### Knowledge, Attitudes, and Clinical Skills

There were three primary outcomes for this study. Sexual health knowledge was assessed using 16 dichotomously scored items created by the research team. The items covered six categories: female sexual health concerns (two items), sexual development and masturbation (three items), sexual orientation (three items), sexual violence (three items), sexuality in middle age (three items), sexual history taking and sexual counseling (two items). Total scores were used for analysis (maximum total score of 16). Sexual health attitudes were assessed using the Sexual Health Education for Professionals Scale (SHEPS) (Ross et al., 2018). The curriculum was designed to build confidence in participants’ knowledge to assess the sexual health of patients, and confidence in their ability to discuss their patient’s sexual health concerns. Each section consists of 37 items where participants rate their confidence from (1) very unconfident to (5) confident. Total scores were used for these analyses (maximum total score of 185 = totally confident). *Sexual Counseling Skills* were assessed by faculty raters assessing the video of student counseling blind to whether the participant was in the intervention or control group and whether the assessment was at baseline or follow-up. Each participant was rated on 10 items assessing their interpersonal communication abilities on a three-point scale (0 = not done; 1 = partially done; 2 = done). Thus, participants could obtain a total of 20 points for each scenario. For medical history taking, six key pieces of information were identified in each case rated on a two-point scale. Participants received a 0 if they did not solicit this information and a 1 if they obtained it. Participants could score 0–6 for the medical history section. To create an aggregated score for each time point, the total scores for the two videos at baseline and two videos at follow-up were summed. Therefore, participants could obtain a total score of 40 for the interpersonal communication scale and a total score of 12 for medical history-taking scale.

#### Sexual Health Beliefs

In addition to the primary outcomes, we investigated the effects of the intervention on *Sexual Health Beliefs* using the *SHEPS* Attitudes section. This comprises 27 items (e.g., “Masturbation is a healthy part of human development”). Participants rate their level of agreement (1 = strongly agree; 5 = strongly disagree), with 13 items being reverse coded. On this section, total scores range from 27 (“liberal” or “open” views) to 135 (“conservative” or “closed to discuss”) views, with 81 considered neutral.

#### Seminar Evaluation of Helpfulness and Acceptability

Immediately following the intervention, intervention arm participants were asked to evaluate the workshop. Thirteen items assessed the workshop (e.g., the workshop was fun; the workshop taught medically accurate information (1 = strongly agree; 5 = strongly disagree). Then, participants were asked to rate each module as helpful to unhelpful (37 items). Sample items include: “lecture on sexual health in midlife and older life,” “videos in Swahili showing how to ask sexual health questions” and “small group discussion about case of sexual orientation.” Cultural and personal acceptability of the curriculum was assessed using seven items; four of which assessed “this workshop was respectful of my …” gender, race and tribal background, religion and religious background, and sexual values and traditions (1 = strongly agree; 5 = strongly disagree), respectively. Three items used a different stem: “This workshop was …” to assess appropriate for Africa, more appropriate for the USA, and appropriate for training health professionals (20 items). A priori, we set 70 percent or more of students evaluating a session positively or as acceptability as evidence of good acceptability.

### Statistical Analysis

The intervention effect was evaluated using a multiple regression analysis. The intervention group was assessed at baseline, immediately post-intervention and follow-up, while the control group was assessed at baseline and follow-up only. Thus, difference scores (follow-up–baseline) were calculated for each outcome listed above and used as the outcome for the regression models. "Intervention” (dummy coded as 0 = control, 1 = intervention) was the primary predictor in the model. To assess whether there was a differential effect of intervention based on demographics, students’ discipline, gender and religious affiliation were included as interaction effects (full model). In the current study, we recruited nursing, midwifery and medical students. Due to the small number of midwifery students, midwives were grouped with nurses for modeling purposes. Thus, discipline was recoded into a variable called “Nursing” (0 = medicine, 1 = nurse/midwife). Similarly, gender was coded as “Female” (0 = male, 1 = female) and religion was coded as “Muslim” (0 = Christian, 1 = Muslim), based on the demographic groups with the most responses (see Table 1 for demographic information). If the interaction effects between Nursing, Female and Muslim with Intervention were not statistically significant, the interactions were removed from the model and the reduced model (without interaction effects but still including the main effects) was fit and interpreted. All analyses were run using R software (v 4.2.1) (R Core Team, 2022).

Of the 412 recruited, one participant did not complete the post-intervention survey and three participants did not complete the three-month follow-up. Furthermore, if participants did not identify as male/female or Christian/Muslim (e.g., responded “Other” or “Prefer Not to Answer”; see Table 1), they were considered missing data and removed from analysis. This resulted in 392 participants for statistical modeling (95% of the total sample).

## Results

The demographic characteristics of the sample are reported in Table 1. To summarize, about two-thirds of the sample were medical students and one-third, nursing or midwifery students, and about evenly split between students in their final year or penultimate year.

Most of the students (*n* = 276; 67.0%)) were male and aged 22–24 years. Almost all (*n* = 382; 92.7%) were single, non-cohabitating. Sexually, most identified as either heterosexual (*n* = 304; 73.8%) or asexual (*n* = 11.9%). Religiously, most identified as Christian (*n* = 349; 84.7%) or Muslim (*n* = 59; 14.3%), and most (*n* = 378; 91.7%) evaluated themselves as moderately to very religious. About a third (*n* = 132; 32.5%) reported no sexual health education prior to starting medical or nursing school. Sources of sexual health information included from the Internet (*n* = 351; 85.2%), peers (*n* = 310; 75.2%), parents/relatives (*n* = 207; 50.2%), through clinical rotations (*n* = 196; 47.6%), patients (*n* = 160, 38.8%), a workshop (*n* = 143; 34.7%) and other (*n* = 122; 29.6%) with the most common write-in being from religious gatherings/teachings.

**Fig. 2:**
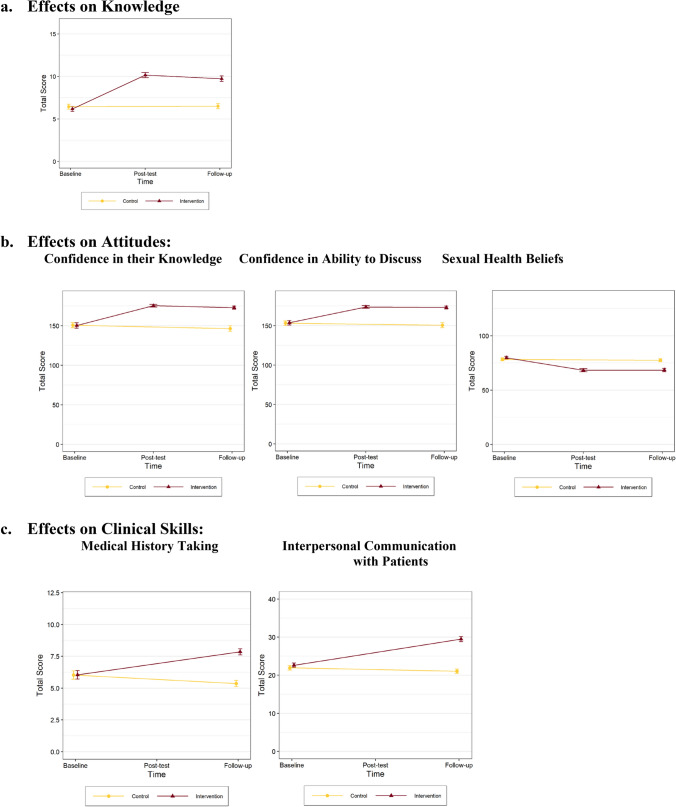
Graphs showing changes in observed mean scores in students’ knowledge, attitudes and clinical skills by intervention group and time (*N* = 408 students)

Table 2 presents the results for the primary outcomes. Based on the full model results for the primary outcomes, there was not a statistically significant interaction effect between Intervention and discipline or gender for knowledge, confidence in discussing or sexual counseling skills (interpersonal communication and medical history taking). Thus, the reduced models for these outcomes are presented in Table 2 and Fig. 2. However, there was a statistically significant interaction between religion and intervention for confidence in having knowledge ($\beta =-21.01, SE=6.53, p=0.001$). Intervention group participants who identified as Muslim showed a smaller increase in their confidence in having sexual health knowledge from baseline to follow-up compared to participants who identified as Christian.

**Table 2:** Results of regression models for primary outcomes

	Estimate ($\beta$)	SE	*CI*_*low*_	*CI*_*high*_	*p*	${R}_{adj}^{2}$
Sexual health knowledge						0.357
(Intercept)	0.16	0.21	− 0.25	0.56	0.44	
Intervention	3.49	0.24	3.02	3.95	< .001	
Nursing	− 0.28	0.25	− 0.77	0.22	0.27	
Female	0.16	0.25	− 0.34	0.66	0.54	
Muslim	− 0.22	0.34	− 0.90	0.45	0.51	
Sexual health attitudes						
*Confidence in ability to discuss*						0.241
(Intercept)	− 2.46	1.74	− 5.88	0.97	0.6	
Intervention	22.00	1.99	18.09	25.91	< .001	
Nursing	− 2.92	2.13	− 7.11	1.27	0.17	
Female	0.96	2.15	− 3.25	5.18	0.65	
Muslim	4.26	2.89	− 1.42	9.95	0.14	
*Confidence in having knowledge*						0.289
(Intercept)	− 5.07	2.31	− 9.61	− 0.53		0.029
Intervention	29.34	3.26	22.94	35.74	< .001	
Nursing	− 4.45	3.41	− 11.16	2.26	0.19	
Female	1.31	3.43	− 5.44	8.05	0.70	
Muslim	14.21	4.75	4.87	23.54	0.003	
Intervention x Nursing	− 1.08	4.81	− 10.53	8.37	0.82	
Intervention x Female	3.64	4.84	− 5.88	13.15	0.45	
Intervention x Muslim	− 21.01	6.53	− 33.85	− 8.17	0.001	
Sexual counseling skills						
*Interpersonal communication (IC)*						0.337
(Intercept)	− 0.15	0.52	− 1.17	0.88	0.78	
Intervention	8.04	0.60	6.87	9.21	< .001	
Nursing	− 2.87	0.64	− 4.13	− 1.61	< .001	
Female	0.67	0.64	− 0.59	1.94	0.30	
Muslim	− 1.10	0.87	− 2.80	0.61	0.21	
*Medical history taking (MHT)*						0.169
(Intercept)	− 0.85	0.25	− 1.34	− 0.37	< .001	
Intervention	2.50	0.28	1.94	3.05	0.006	
Nursing	0.67	0.30	0.08	1.27	0.028	
Female	− 0.12	0.31	− 0.73	0.48	0.68	
Muslim	0.13	0.41	− 0.68	0.94	0.76	
Sexual health beliefs						0.191
(Intercept)	− 0.59	0.91	− 2.38	1.21	0.52	
Intervention	− 1.21	1.04	− 12.26	− 8.16	< .001	
Nursing	− 0.14	1.12	− 2.34	2.06	0.90	
Female	− 0.89	1.13	− 3.10	1.33	0.43	
Muslim	0.56	1.52	− 2.42	3.55	0.71

There was a statistically significant positive effect of Intervention for every primary outcome both in crude analysis and after controlling for discipline, gender and religion. The intervention group showed larger gains (from baseline to follow-up) in sexual health knowledge ($\beta =3.49, SE=0.24, p<0.001$), confidence in their ability to discuss ($\beta =22.00, SE=1.99, p<0.001$), confidence in having knowledge ($\beta =29.34, SE=3.26, p<0.001$), interpersonal communication skills ($\beta =8.04, SE=0.60,\, p<0.001$) and medical history-taking skills ($\beta =2.50, SE=0.28, p<0.001$) compared to the control group (regardless of their demographic background). Compared to the other covariates in the model, intervention had the largest effect on difference scores (largest regression estimates). In addition, the participants became more open in their beliefs about sexual health topics ($\beta =-10.21, SE=1.04, p<0.001$) compared to the control group, regardless of their demographic background. Again, intervention had the largest effect on difference scores.

Almost all (96.1%) participants rated the curriculum as personally acceptable, as communicating clearly about sex and sexual health (100%), as appropriate training for future health professionals (98.0%) and as culturally respectful in Africa (76.6%). Almost all (98.5%) agreed or strongly agreed that they would recommend the training to their fellow students, with the remainder being neutral. The most culturally sensitive topics, namely masturbation, sexual orientation and gender diversity, and sexuality in older age were evaluated as helpful by 99.5%, 96.6% and 98.3% of students, respectively. Effects were similar for both nursing/midwifery and medical students.

## Discussion

The main finding of this trial is that a comprehensive sexual health curriculum intervention was successful in increasing students’ knowledge of sexual health, improved their confidence in addressing their patient’s concerns and to discuss these with patients; and in building clinical skills to assess and treat patients. We note participants’ sexual beliefs also became more liberal, possibly reflecting a greater underlying comfort and openness with addressing sexual health concerns. Against the notion that sexual health education is culturally taboo in socially conservative countries, most students evaluated the intervention as acceptable and culturally appropriate across multiple dimensions.

To the best of our review, this is the first randomized controlled trial of a sexual health curriculum for health students, as well as the first to be conducted in Africa. We highlight that the observed changes were all moderate to large in size, and for clinical skills, observable both by self-report and to blinded raters.

There are several limitations to consider when interpreting these results. First, for ethical reasons, we relied on volunteers to participate in this study. Some students who may have been opposed to a sexual health curriculum may have self-selected not to participate. Second, this study was conducted during the students’ long vacation and the curriculum was presented as a special workshop. Results may vary if the curriculum is integrated into a regular curriculum. Third, the MUHAS-based team spent two–three years conducting the formative research for this study, designing and refining the curriculum, and piloting the materials. Other faculty with less experience may not produce as strong effects, especially if they have less training in teaching sexual health. Fourth, these results were focused on the short-term effects of the curriculum and no observations of the effects of the curriculum on actual patients were undertaken. Fifth, this curriculum was tailored to the needs of health students at one university in Tanzania who were training to become clinicians. We do not know the generalizability of these findings to medical, nursing and midwifery students at other institutions or in other countries in Africa nor the appropriateness of the curriculum for health students training for nonclinical careers (e.g., epidemiologists, pharmacists, health educators).

Strengths of this study include a rigorous controlled evaluation design, excellent retention, assessment of clinical skills by experienced clinicians blind to both study condition and time, and reporting of results that were both statistically significant and educationally meaningful. The results were equally strong for both nursing/midwifery and medical students. The key implication is that health universities should consider adding a comprehensive sexual health curriculum as an important component of nursing/midwifery and medical students’ training.

We highlight the location of study in Tanzania to be both a strength and a weakness. As a strength, when considering generalizability to other universities, we see no reason why these results should not generalize to other parts of Africa and to low- and middle-income countries globally, especially those where sexual health challenges are substantial. Given Tanzania is a socially conservative country (Oxford Analytica, 2018), and most students in this study identified as moderately to strongly religious, the evaluations of good cultural acceptability suggest that this curriculum should work across most other countries and settings. As a weakness, we do not know if replication of this study in a high-income country would produce similar or different results. In countries where students universally receive comprehensive sexual health education and have more Internet access to sexual health topics than in Tanzania, the effects of the intervention, at least on knowledge and attitudes, might be diminished. Regardless of whether the country is high, moderate or low income, the need to train health students in sexual health is clearly still important and all the evidence of this study is that it produces better clinicians.

In some countries, including the USA, sexual health education is a controversial topic (Dodge et al., 2008; Irvine, 2004; Wiley et al., 2020), although less so for nursing, midwifery and medical student). Thus, acceptability is a crucial construct to measure. Only one participant dropped out during the workshop (with the reason stated because of illness not withdrawal from the intervention). Across all dimensions of acceptability, between 71.1 and 100% participants rated this sexual health curriculum as acceptable, including 98.5% of students who stated they would recommend the workshop to fellow students. And we note that the sessions which included the most culturally controversial topics such as masturbation, homosexuality and older persons being sexually active were almost universally evaluated as helpful (Rosser et al., 2022).

The finding that sexual health education led to the students adopting more liberal attitudes was unanticipated. While it is consistent with studies from the 1970s, those studies deliberately set out to desensitize students “to stressful and anxiety reactions to sexual stimuli” in order to “develop a more tolerant attitude toward sexual beliefs, attitudes and behaviors of others” (Mims 1976). We speculate that providing accurate and relevant sexual health content improves students’ knowledge while listening to panels of patients likely encourages empathy. These in turn build confidence, while the practice of skills builds skills. Together, improved knowledge, confidence, empathy and skills lead to a reassessment of beliefs and more openness, comfort and sensitivity to the challenges patients have. This is similar to other areas of skills development such as driving a car or preparing a meal. Initially, people may have some trepidation (based on an awareness of how little they know) and negative beliefs (e.g., this is hard), only to replace these with greater openness and more relaxed attitudes once they have achieved more knowledge, confidence in their abilities and actual skills.

We identify three directions for future research. First, now that the short-term effects of such education have been established, it will be important to study the durability of effects and ideally the effects on real patients in the clinical setting. Second, the logical progression of a successful Phase III efficacy trial is a Phase IV/V dissemination trial to replicate the curriculum’s effectiveness at other universities. Third, given these results were in a low income country, replication to test effects in a high-income country would address the question whether sexual health education in high-income countries produces similar results.

This is the first evidence-based sexual health curriculum for health students with proven efficacy. In the next phase of study, we will develop training materials so that colleagues can access the curriculum and be trained in its delivery. Second, we will develop a training program for faculty who may lack sexual health expertise so they can develop competency in teaching sexual health.

This study provides definitive evidence that sexual health education of nursing/midwifery and medical students produces more competent clinicians and that training in sexual health is culturally acceptable, needed and effective for nursing, midwifery and medical students in Tanzania. Now that the effects of such training have been established, our chief recommendation is that those responsible for the training of nursing, midwifery and medical students should consider adding a sexual health curriculum as a standard part of their students’ education.

## Data Availability

During the study, a deidentified dataset and data dictionary are available to other researchers upon request to the corresponding author. At the end of the study in July 2023, these materials will be archived at the Data Repository of the University of Minnesota.
